# What is your diagnosis?

**DOI:** 10.4274/jtgga.2018.0061

**Published:** 2018-06-04

**Authors:** Gülşah Aynaoğlu Yıldız, Metin İngeç, Ömer Erkan Yapça

**Affiliations:** 1 Department of Obstetrics and Gynecology, Atatürk University School of Medicine, Erzurum, Turkey

A 28-year-old pregnant patient in her 27^th^ week of pregnancy with G2P0 was referred to our clinic due to the detection of a mass in the baby’s heart. In an echocardiographic examination, a 13x6 mm hyperechogenic mass that was moving together with the pulmonary valve, and hypertrophy in the right ventricle were observed (Figure 1). No chromosome anomalies were detected in the genetic analysis performed via cordocentesis. The pregnant patient was sent to another hospital where she delivered the baby. The baby underwent surgery on the 20^th^ day and died on the 10^th^ postoperative day.

## Answer

Primary cardiac tumors are mesenchymal or hamartomatous nodules originating from the heart layers located in the heart or pericardium. These tumors are rarely seen, but the diagnostic rates are increasing with the recent use of echocardiography. Metastatic cardiac tumors have also been described, but they are even rarer. Rhabdomyosarcomas are usually encapsulated and numerous. These are the most common, accounting for three-quarters of the cardiac masses detected in fetuses and newborns. Ninety percent of them are multiple and they tend to grow into cavities. The most common complications are hydrops fetalis, ventricular outflow obstruction, arrhythmia, and cardiac shock. Surgical treatment should be undertaken if it leads to mechanical stenosis in the heart or causes life-threatening arrhythmia (1). Intrauterine diagnosis is quite difficult. Most diagnoses are postpartum, ranging from 4.3 months to 18 years on average (2). The second most common type is teratomas, which form 15% of cardiac tumors, are single and encapsulated, and tend to grow in the pericardial cavities. Fibroma, hemangiomas, and myomas (less than 5%) are even less common. The rarest are lipomas. Myxomas are the most common benign tumors in adults and constitute approximately 50% of masses in the heart. However, very few cases have been reported during the neonatal period. Diagnosis is usually postpartum, but intrauterine diagnosis can be made in very few cases (3). These appear as echogenic, long pedunculated lesions in the 23^rd^ gestational week with echocardiography. More than 90% are benign. 

We present the following case because it is still difficult to diagnose intrauterine primary cardiac tumors. 

The baby was diagnosed as having myxoma. A total of 32 fetal cardiac myxoma cases have been reported in the literature. Our case is the 33^rd^. It is usually seen in the left ventricle and the rarest site is the left atrium. In our case, the mass was located in the right ventricle. The localization and size of the mass may be predictive for prognosis. Echocardiography is the most reliable method for diagnosing myxoma as it is in diagnosing other cardiac tumors. It was reported that in the echocardiography performed in the 23^rd^ gestational week, the myxoma was seen as a soft and echogenic mass with a long pedunculated lesion (4). Although standard therapy is surgical resection, an appropriate approach to embolism risk should be identified. Small and unobstructed immobile masses may be left and monitored, but large tumors bearing embolism risk and obstruction should be treated surgically. In utero open surgery in immature fetuses with hydrops can be an option. Small tumors, especially those on the heart wall and not protruding into the cavity, may not be easy to recognize. In particular, masses in the papillary muscle, seen as echogenic foci, may mimic rhabdomyomas. Rhabdomyomas are usually multiple nodular and echogenic masses seen in the atrium or ventricles. Myxomas are usually located in the atrium and move with cardiac contractions. 

Growing tumors can cause cardiac tamponade, heart dysfunction, hydrops fetalis, and death. A different feature of cardiac rhabdomyomas from other primary cardiac tumors is tuberous sclerosis and its genetic background. Tuberous sclerosis was detected in approximately 76% of cases in which rhabdomyomas were detected. Most cases of tuberous sclerosis can be recognized by deletion mutations in the TSC2 and PKD1 genes (5). Fetal cardiac tumors may generally be recognized during the 20^th^-30^th^ weeks of gestation. The earliest reported case week is 17. The most important method of diagnosis is echocardiography. Findings such as cardiomegaly, pericardial effusion, arrhythmia, and ventricular outflow obstructions suggest a tumor.

In conclusion we do not think it is appropriate to present termination as an option for pregnancies with primary fetal cardiac tumors because of the difficulty of intrauterine diagnosis.

## Figures and Tables

**Figure 1:**
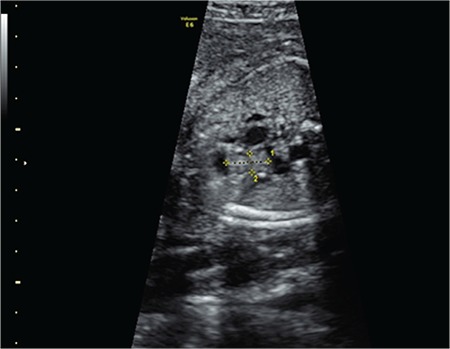
Ultrasonographic image of the mass
